# A Case Report of Bilateral Adrenal Gland Stereotactic Body Radiotherapy to Manage Hypercortisolemia in a Patient With Ectopic Adrenocorticotropic Hormone (ACTH) Production From a Metastatic Pancreatic Neuroendocrine Tumor

**DOI:** 10.7759/cureus.57852

**Published:** 2024-04-08

**Authors:** Said Al Saifi, Irena Druce, Michael Vickers, Kristopher Dennis

**Affiliations:** 1 Department of Radiation Oncology, The Ottawa Hospital and the University of Ottawa, Ottawa, CAN; 2 Division of Endocrinology and Metabolism, Department of Medicine, The Ottawa Hospital and the University of Ottawa, Ottawa, CAN; 3 Division of Medical Oncology, Department of Medicine, The Ottawa Hospital and the University of Ottawa, Ottawa, CAN

**Keywords:** stereotactic body radiotherapy, pancreatic neoplasms, neuroendocrine tumors, hypercortisolism, ectopic acth syndromes, adrenal glands

## Abstract

A 63-year-old woman presented with hypokalemia, hypertension, weight gain, limb edema, and tremors. She was diagnosed with Cushing syndrome, with a 24-hour urine cortisol level of 41,013 nmol/day. Investigations revealed a grade 2 pancreatic neuroendocrine tumor with extensive hepatic metastases. Owing to excessive adrenocorticotropic hormone production from her disease, her hypercortisolemia and Cushing symptoms worsened despite ketoconazole, somatostatin analogs, and right liver lobe chemoembolization. Stereotactic body radiotherapy (SBRT) at a dose of 39 Gy in three fractions was administered to her bilateral adrenal glands in the hope of reducing her cortisol levels and improving her symptoms. Her 24-hour urine cortisol levels decreased following SBRT, but not rapidly enough; her clinical condition continued to deteriorate, and she died 21 days after treatment. SBRT was not effective as an urgent intervention in this setting; a greater latency to realize a response is likely necessary.

## Introduction

Pancreatic neuroendocrine tumors (PNETs) account for approximately 2% of pancreatic tumors [1]. PNETs can be classified histologically as well-differentiated and poorly differentiated and clinically as functioning and nonfunctioning. Functioning tumors represent approximately 30% of PNETs and include insulinomas, gastrinomas, glucagonomas, somatostatinomas, vasoactive intestinal peptide tumors, and adrenocorticotropic hormone (ACTH)-producing tumors [2,3].

ACTH-secreting PNETs are uncommon; they are usually associated with liver metastases that develop prior to patients noticing symptoms, and they can be associated with a poor prognosis [4-7]. High ACTH levels can result in hypercortisolemia and Cushing syndrome, with its attendant neuropsychiatric, metabolic, dermatologic, musculoskeletal, and cardiovascular symptoms [8].

Initial management of Cushing syndrome can include ketoconazole and somatostatin analogs [9-12]. If symptoms persist despite pharmacologic therapy, local therapy options can be considered. In the setting of a metastatic PNET, these can include resection of the primary tumor and/or bulky liver metastases, chemoembolization of liver metastases, adrenalectomy, and adrenal radiofrequency ablation.

If such options are not possible or prove ineffective, an alternative nonsurgical local treatment could be adrenal ablation with stereotactic body radiotherapy (SBRT). Stereotactic radiotherapy is a standard treatment option for functioning pituitary tumors, including those that secrete ACTH [13,14]. To our knowledge, however, the use of adrenal SBRT to manage hypercortisolemia secondary to an ACTH-secreting PNET has not been reported. There is a known iatrogenic risk of adrenal insufficiency months following bilateral adrenal tumor irradiation otherwise [15] (e.g., in the setting of aggressive treatment for oligometastatic cancers); however, whether the latency to insufficiency following planned comprehensive bilateral adrenal SBRT would be too long to help in the setting of worsening Cushing syndrome symptoms is an open question.

We report here a case of attempted symptom control with bilateral adrenal SBRT in a patient with treatment-refractory Cushing syndrome secondary to an ACTH-secreting metastatic PNET.

## Case presentation

**Figure 1:**
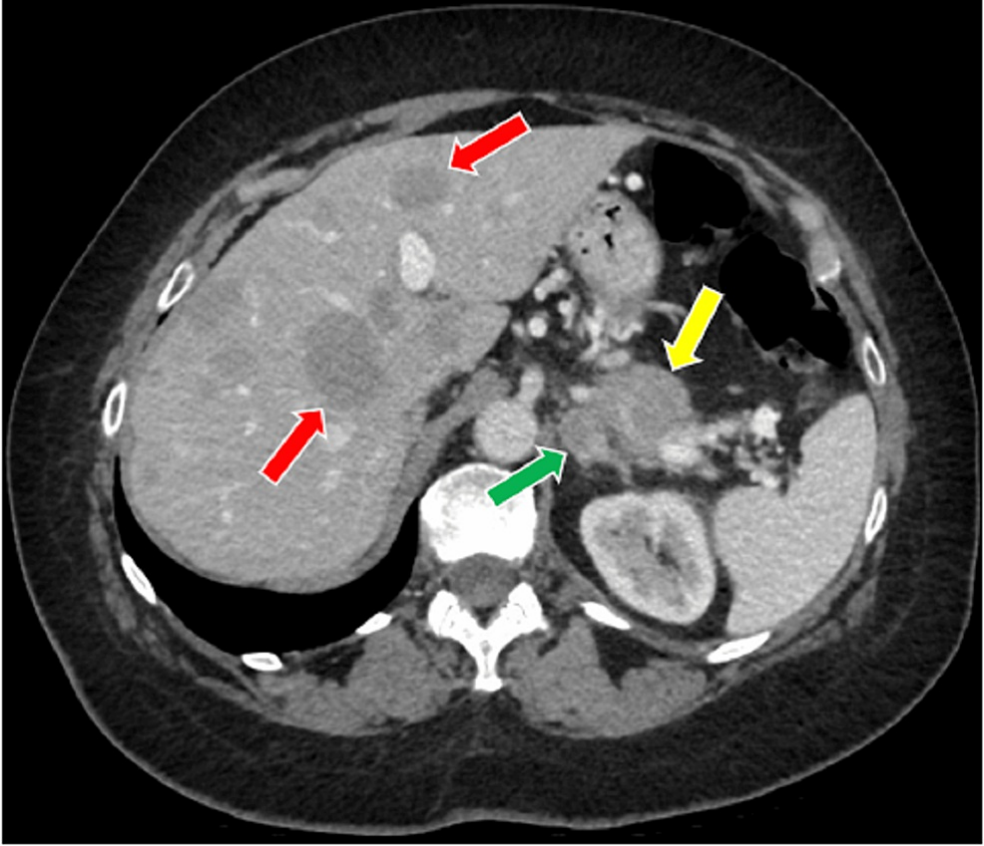
CT axial plane image of left adrenal mass (green arrow) adjacent to primary pancreatic mass (yellow arrow) and hepatic metastases (red arrows)

A previously healthy 63-year-old woman presented with a three-week history of unexplained hypertension, weight gain from 183 to 200 pounds, bilateral upper limb edema, and whole-body tremors occurring approximately six times daily and lasting between five and 10 minutes. On assessment in the emergency room, a blanchable maculopapular rash was seen on her neck, arms, and legs. Her bloodwork showed hypokalemia (2.5 nmol/L, reference (ref) range: 3.5-5.1) with metabolic alkalosis, an elevated ACTH (105 pmol/L, ref range: 1.6-13.9), hypercortisolemia (2,587 nmol/L, ref range: 68-327), and mildly elevated liver enzymes (aspartate aminotransferase: 39 U/L, ref range: 12-29; alanine aminotransferase: 65 U/L, ref range: 8-33). She was admitted with suspected Cushing syndrome, and a 24-hour urine cortisol test confirmed this with a level of 41,013 nmol/day (ref range: 60-415). CT of the head, chest, abdomen, and pelvis showed a 3.5-cm pancreatic tail mass suspicious for a primary tumor, a 1.7-cm left adrenal nodule, and innumerable hepatic metastases (Figure 1). An ultrasound-guided biopsy of a liver metastasis confirmed a grade 2 neuroendocrine tumor with a Ki67 of 6% and cells staining positive for cytokeratin 8/18, chromogranin A, and inhibin and negative for synaptophysin and P40.

The patient was treated with potassium replacement and spironolactone to manage the apparent mineralocorticoid excess, ketoconazole to reduce cortisol production, and once pathology confirmed a PNET, short-acting and eventually long-acting somatostatin analogs were added. However, her symptoms due to hypercortisolemia persisted, and she ultimately agreed to a right liver lobe portal vein embolization in the hope of reducing the amount of ectopic ACTH being secreted from her hepatic metastases. She was discharged the day following embolization, approximately three weeks from her initial admission, with her 24-hour urine cortisol level reduced from baseline at 22,982 nmol/day (Table 1).

**Table 1:** Serum and 24-hour urine cortisol levels over time KTC, ketoconazole; SBRT, stereotactic body radiotherapy; SSA, somatostatin analog

Time point	Description	Serum cortisol (nmol/L)	24-hour urine cortisol (nmol/d)
Day 0	Initial presentation and admission	2,587	
Day 2			41,013
Day 19	After KTC and SSAs		22,982
Day 23	Discharge	3,448	
Day 39	18 days after liver embolization		14,216
Day 45	Readmission	6,127	
Day 50	4 days prior to SBRT	6,507	
Day 59	1 day after SBRT	5,855	
Day 60	2 days after SBRT	4,643	
Day 61	3 days after SBRT	5,523	
Day 64	6 days after SBRT	6,193	
Day 67	9 days after SBRT	7,595	
Day 68	10 days after SBRT		10,300
Day 70	12 days after SBRT	8,206	

**Figure 2:**
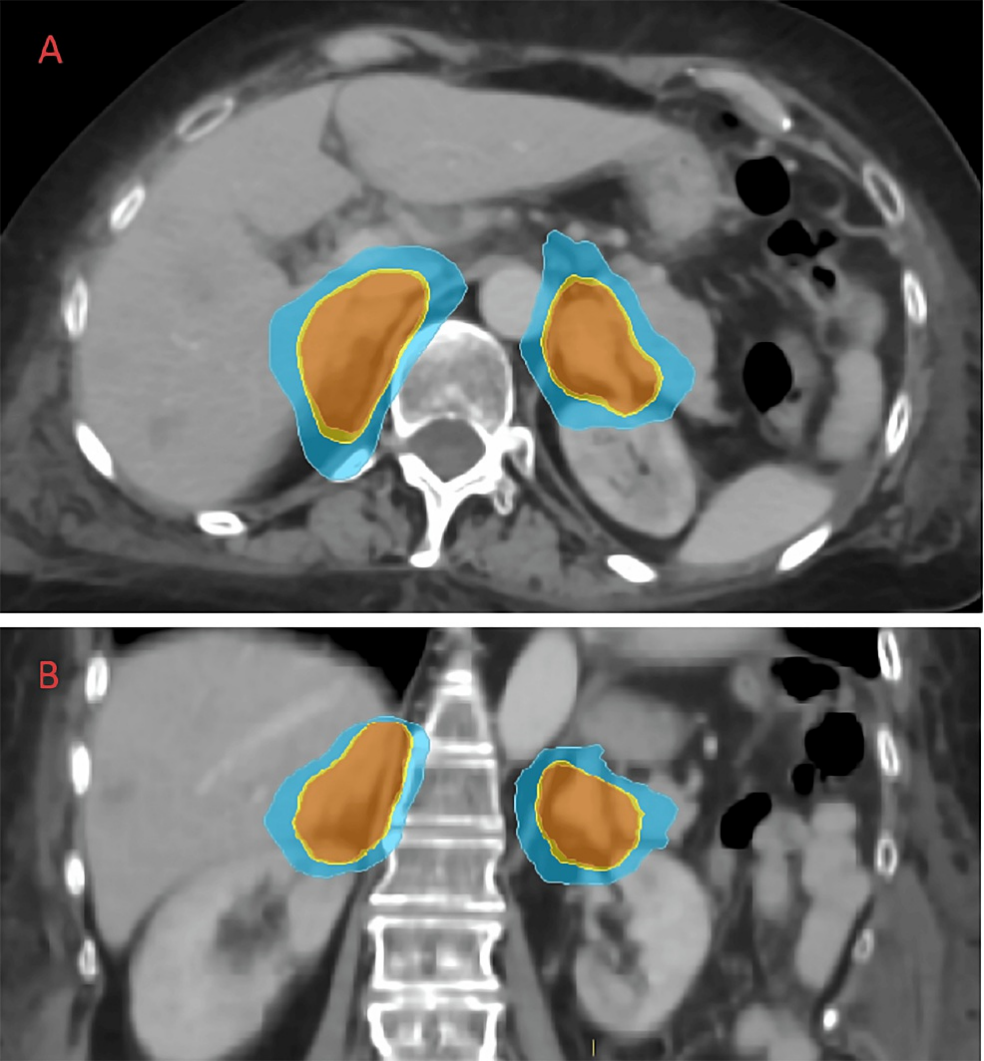
Radiotherapy treatment planning CT axial plane image (A) and coronal plane image (B) showing isodose levels overlying the adrenal glands: 3,900 cGy/100% (orange), 3,705 cGy/95% (yellow), 3,000 cGy/77% (green), and 2,730 cGy/70% (blue)

The patient was admitted again approximately three weeks later with persistent symptoms of Cushing syndrome, including significant depression. Her 24-hour urine cortisol level was 14,216 nmol/day. In the hope of further improving her hypercortisolism, she agreed to a course of SBRT for both adrenal glands at a dose of 39 Gy in three fractions, administered on consecutive days (Figure 2). She tolerated treatment well without troublesome acute toxicities. In tracking her biochemical response to treatment, we observed a difference between serum and urine cortisol levels (Table 1). Four days prior to SBRT, her serum cortisol was 6,507 nmol/L. It reached a nadir of 4,643 nmol/L two days after SBRT completion but then rose to 8,206 nmol/L 12 days after SBRT. By comparison, her 24-urine cortisol level (a more reliable indicator of treatment response) 10 days after SBRT had dropped from its pretreatment level to 10,300 nmol/day. Unfortunately, despite this incremental improvement, by that time her depression had worsened, she was having intermittent bouts of symptomatic hypotension, she had developed significant upper and lower limb edema, and her blood cultures had grown *Escherichia coli *from a presumed urinary source. A decision was made to focus on comfort care alone, and she died 21 days after SBRT, less than three months after her initial presentation. An autopsy was declined by the family.

## Discussion

This is the first case report of which we are aware that describes the use of bilateral adrenal SBRT in the hope of controlling symptoms from hypercortisolemia in the setting of an otherwise treatment-refractory PNET causing excessive ACTH production.

The treatment of ectopic ACTH can be challenging, and management should be individualized and guided by multidisciplinary collaboration. Guidelines and expert opinions exist for the management of Cushing syndrome due to ectopic ACTH, but owing to the very heterogeneous affected patient phenotypes, no simple algorithm can be followed [16-18]. In general, if medical therapy cannot adequately control the symptoms of a metastatic PNET, local therapy options such as adrenalectomy or adrenal radiofrequency ablation can be considered, along with surgical resection of the primary and/or hepatic metastases or chemoembolization of extensive areas of hepatic metastases. Given our patient’s poor performance status and personal wishes, along with the extensive distribution of her disease, after her chemoembolization procedure, nonsurgical therapies were pursued. Prognostic models for affected patients could potentially assist in clinical decision-making, and some cohort studies have found that certain features such as hypokalemia and acute onset are associated with worse outcomes [17,18], but again, owing to the heterogeneous patient presentations, formal prognostication tools are still lacking.

The SBRT prescription administered to our patient (39 Gy in three fractions; biologically equivalent dose (BED10) of 89.7 Gy) was in line with those reported in a series of SBRT regimens of different durations and doses for adrenal metastases elsewhere [19,20]. Owing to her declining performance status, a short fractionation schedule was desired. A systematic review of 39 studies with 1,006 patients who received SBRT for adrenal metastases (66% of patients had lung cancer) suggested a possible dose response with regimens with a BED10 >100 Gy potentially providing better local control; however, adrenal toxicities were rarely addressed in the contributing studies, with only five total patients from two studies being listed as developing grade 2 adrenal insufficiency [16]. Whether higher BED regimens within the SBRT range predict greater impairment of adrenal function is an open question.

A single-institution study published after the systematic review described findings from 56 patients who had undergone adrenal SBRT for metastatic disease and had ongoing assessments by an endocrinologist [15]. Among these carefully monitored patients, 80% of those who received treatment for both glands (or their solitary remaining gland) developed primary adrenal insufficiency with a median time of onset of six months following SBRT (range: 0.5-20.4 months). This suggests that, by extrapolation, using bilateral SBRT for symptom control in a setting such as the one we report here may not, on its own, provide rapid improvements in hypercortisolemia and Cushing symptoms. Continued bridging medical therapy is likely necessary while waiting for the effect of SBRT to be realized, and using SBRT earlier in a patient’s treatment course should be considered.

Our case also highlights the importance of monitoring treatment response not only with point-of-care serum cortisol levels but also with more reliable 24-hour urine cortisol levels. Despite declining urinary levels after both the liver embolization and later SBRT, in the 21 days following SBRT during which our patient was living, serum levels steadily increased. Monitoring only serum levels can lead to misinterpretations of treatment effectiveness in this setting.

## Conclusions

We report here a unique case of bilateral adrenal SBRT administered in the hope of controlling symptoms from hypercortisolemia in the setting of an otherwise treatment-refractory metastatic PNET causing excessive ACTH production. Our patient’s cortisol levels improved over time following sequential therapies including SBRT but remained significantly elevated, leading to clinical decline and death 21 days following SBRT. Practitioners with similarly affected patients should likely expect a delayed response to SBRT in this setting and not rely on it solely to induce rapid clinical improvements.
